# Chronic Pain-Induced Depression: A Review of Prevalence and Management

**DOI:** 10.7759/cureus.28416

**Published:** 2022-08-25

**Authors:** Roja T Meda, Surya P Nuguru, Sriker Rachakonda, Shravani Sripathi, Mashal I Khan, Naomi Patel

**Affiliations:** 1 Research, Narayana Medical College, Nellore, IND; 2 Internal Medicine, Kamineni Academy of Medical Sciences and Research Centre, Hyderabad, IND; 3 Surgery, Bogomolets National Medical University, Kyiv, UKR; 4 Surgery, Bhaskar Medical College, Hyderabad, IND; 5 Internal Medicine, Khyber Girls Medical College, Peshawar, PAK; 6 Research, Smt. Nathiba Hargovandas Lakhmichand (NHL) Municipal Medical College, Ahmedabad, IND

**Keywords:** chronic pain and depression, chronic pain induced depression management, pharmacological management of chronic pain, prevalence of chronic pain, management of depression in chronic illness, depression in chronic illness, depression, chronic pain induced depression, chronic pain

## Abstract

Chronic pain is ongoing pain that has persisted beyond standard tissue healing time along with comorbidities such as depression. This article discusses studies that have shown the prevalence of chronic pain and chronic pain-induced depression and explained methods of prevention for these conditions. The molecular mechanisms such as monoamine neurotransmitters, brain-derived neurotrophic factor, inflammatory factors, and glutamate that are similar in chronic pain and depression have also been discussed. This article reviews the methods of management that utilize the identification of these molecular mechanisms to treat this condition further. It also emphasizes the importance of the awareness of chronic pain-induced depression for the upcoming advances in the subject of mental health.

## Introduction and background

According to the International Association for the Study of Pain, chronic pain is pain that has persisted beyond normal tissue healing time (which, in the absence of other factors, is generally taken to be three months) [1]. This pain cannot be managed and cured with standard biomedical measures and, thus, treatment for such patients comprises long-term resources to make their suffering more tolerable [2].

The importance of knowing epidemiology should not be undermined since it is key to understanding chronic pain [1]. A lot of risk factors determine the prevalence and incidence of the disease, as shown in Table 1 [1].

**Table 1:** Risk factors for chronic pain

	Factors
Demographic	Age, ethnicity, socio-economic background, gender
Lifestyle and behavior	Physical activity, smoking, alcohol, eating habits
Clinical	Mental health, weight gain, surgical and medical interventions
Other	Preconceived notions about pain, history of violence or abuse

The cause of chronic pain is usually not due to a single event but to a consolidation of factors. The most critical risk factor that can be modified is health-related behavior [1]. Chronic pain develops due to nerve injury or transient tissue, causing constant modifications in cells predominantly upregulated by neuropathic and inflammatory pain. Limited research has shown that alterations in chromatin structure caused by injury form changes in the function of neurons and gene expression, which can lead to symptoms such as depression and anxiety [3]. Epigenetic modifications such as deoxyribonucleic acid (DNA) methylation and histone acetylation have been involved in gene expression. These modifications are altered by drugs, environmental toxins, lifestyle changes, and psychological stress [4].

Primarily, a self-report is used as a method of assessment due to pain being a personal experience. Assessments can be done using various techniques such as visual analog scales (VAS), numerical rating scales, and categorical measuring scales. The most used method today is the numerical rating scale due to its ease of administration and patient compliance [5].

Drugs such as antidepressants and pain medications can be used as treatment, but there is not much evidence that suggests their efficacy. However, there is a rise in the use of nonpharmacological methods such as therapy, acupuncture, hypnosis, and exercise [6].

A study by Magni et al. states that the chances of patients with chronic pain having depression criteria are three times more than patients without chronic pain [7]. Due to the increasing prevalence, it is essential to explore the relationship between chronic pain and depression. There is also a lack of mental health awareness in society, which becomes a problem for patients; for example, the elderly suffering from conditions such as depression. There is a relationship between cognitive impairment and depression that needs to be explored [6]. This review article aims to highlight the prevalence of chronic pain-induced depression and the importance of knowing the prevention and treatment of this condition to increase awareness.

## Review

Impact of chronic pain on depression

Pathogenesis of Chronic Pain

Chronic pain can persist even in cases with no apparent pathological trigger and lasts long after the original trauma has healed. These characteristics of chronic pain make it difficult to understand and are the reason for many research studies being done on it for better insight into the condition [8]. Pain regulation occurs due to the descending inhibitory fibers becoming activated and prevention of pain signal transmission in the spinal cord. The perception of pain is facilitated by the third-order neurons. Neuropathic, nociceptive, and neuroplastic processes are what make pain a complex mechanism [9]. Skin fibers with molecular sensors that can identify peripheral stimuli are known as nociceptors. They relay signs of pain through the dorsal root ganglia to the dorsal horn of the spinal cord; this is where pain begins through the activation of nociceptors [10]. Nociceptive pain is mostly seen due to malfunction in nociceptors involved in transduction and occurs due to peripheral tissue injury, and in chronic pain, peripheral nociceptors that persistently transmit painful impulses even after healing of the initial injury can be seen [9]. Inflammation is another process that plays a pivotal part in chronic pain disorders such as back pain, arthritis, and headaches [9,10]. Inflammatory mediators are released by immune cells in inflammatory tissue innervated by peripheral nerves. This response causes sensor neurons to activate action potentials, increasing excitability and transduction. Immune cells are activated in the spinal cord and dorsal root ganglia, where they regulate pain sensitivity or injure central transmission [9].

Pathophysiology of Chronic Pain-Induced Depression

Various molecular mechanisms are involved in both chronic pain and depression. Monoamine neurotransmitters, brain-derived neurotrophic factor, inflammatory factors, and glutamate and its receptor subtypes are some of such molecular mechanisms [11]. The dopaminergic system of the midbrain affects the neuroplastic alterations of chronic pain and depression. The persistence of chronic pain has the ability to damage dopamine (DA) activity in the midbrain. The protein D2R is the DA receptor that contributes to the development of depression, and as a result of decreased DA levels, there is, in turn, reduced D2R expression. A hypothesis suggests that decreased levels of monoamine neurotransmitters (MN) in the central nervous system increase the chances of developing depression [11].

Another mechanism seen to affect chronic pain and depression is inflammatory factors that cause changes in the functional areas that affect depression. They act through the blood-brain barrier and cause alterations in neurotransmitter metabolism and neuroendocrine function [ 11]. One of the primary excitatory neurotransmitters in the central nervous system is glutamate. Glutamate and its subtypes (N-methyl-D-aspartic acid receptor, α-amino-3-hydroxy-5-methyl-4-isoxazolepropionic acid receptor) is also seen to be involved in the process of chronic pain and depression development. On discovering neuroplastic changes that are common to both chronic pain and depression, therapeutic drugs can be utilized or developed targeting specific areas common to both conditions for precise treatment [11].

Epigenetic modifications have also been seen to play a role in the comorbidity of chronic pain leading to depression. These modifications cause a change in the structure of chromatin, which regulates the ability of transcription factors to reach promoter regions on DNA [12]. The mechanisms used for this process include DNA and histone methylation, histone acetylation, ubiquitination, and phosphorylation [12]. A study done by Descalzi et al. on mice showed that histone deacetylase (HDAC) inhibitors that were used showed an increase in HDAC5 levels in the nucleus accumbens and periaqueductal gray matter in mice that were exhibiting depressive-like behaviors in the case of spared nerve injury [12]. The decrease of HDAC5 caused a significant reduction in the depressive-like behaviors of the mice. MicroRNAs that focus on targeting HDAC factors in neuronal and non-neuronal cells also play an essential part in gene regulation. Along with epigenetic aspects, the role of microRNAs is also considered in the comorbidity of chronic pain-induced depression [12].

Adult neurogenesis is another mechanism that has not been completely elucidated in chronic pain-induced depression, although they are still being assessed as helpful. Adult neurogenesis is tested by administering a thymidine analog bromodeoxyuridine (BrdU), which helps identify cells that are active in DNA replication and hence proliferate [12]. This method is useful in showing a connection between chronic pain-induced depression and alteration of neurogenesis in the adult hippocampus. Duric and McCarson conducted a study that involved the dentate gyrus of rats exhibiting BrdU-positive cells, which decreased significantly after long periods of stress or inflammation [13].

Epidemiology of chronic pain

According to the Dictionary of Epidemiology sponsored by the International Epidemiological Association (IEA), the study of the distribution and determinants of health-related states or events in specified populations and the applications of this study to control health problems is known as epidemiology [14]. A good understanding of chronic pain epidemiology will help provide information for better prevention and clinical management of the condition. Depression, cerebrovascular disease, traffic accidents, and coronary heart disease are the four main conditions predicted by the World Health Organization to contribute to the global disease burden by 2030 [15]. A crucial association that should be noted is that chronic pain is a comorbidity for all these conditions. But in recent times, chronic pain is not only acknowledged as a comorbidity but also as a condition of its own with many risk factors [15].

In an epidemiological study in India to identify the prevalence of chronic pain, a telephone survey was conducted with 5004 participants from eight cities [16]. The prevalence of chronic pain according to this study was 13%, and the most prominent areas for pain were the knees, legs, and joints. It was also noted that the participants suffering from chronic pain found it difficult to maintain connections with family and friends and to maintain an independent lifestyle [16].

Prevalence of Depression in Chronic Pain Patients

Depression is characterized as a mood disorder by the International Classification of Diseases [17]. The characteristics of depression are decreased energy, persistent sadness, declining interest in daily activities, shifts in sleep and appetite, and having suicidal thoughts or tendencies for a duration of at least two weeks. Although depression generally occurs in individuals predisposed to it genetically with a combination of social factors, it is also closely associated with chronic medical diseases [17].

Of the 42 studies done on the prevalence of major depression in patients suffering from chronic pain, 31 studies are based on chronic pain patients [7]. Table 2 summarizes the prevalence rates estimated from these studies of patients in various clinics suffering from pain who were identified with depression [7].

**Table 2:** Prevalence of depression in patients with chronic pain

Clinic	Percent prevalence
Pain clinic/inpatient pain programs	52% (1.5%-100%)
Psychiatric clinics	38% (6%-64%)
Orthopedic clinics/rheumatology clinics	56% (21%-89%)
Dental clinics focusing on facial pain	85% (35%-100%)
Gynecology clinics focusing on chronic pelvic pain in laparoscopy patients	13% (12%-17%)
Population based settings	18% (4.7%-22%)
Primary care clinics	27% (5.9%-46%)

The lifetime prevalence of depression in chronic pain patients was also estimated in two other studies. The result increased from 12% to 32% in the first study and 32.4% to 56.8% in the second study [7]. Several studies showed that along with the increase in prevalence, as pain worsened, the number of symptoms, duration or severity, and the risk of depression also increased [7].

To investigate the incidence of depression and the association of chronic diseases with pain, Ma et al. conducted a cross-sectional study in China with a study population of 15,213 individuals from the 2015 China Health and Retirement Longitudinal Study [18]. The independent variable was chronic disease, and the degree of pain was one of the factors assessed. The result was that increased severity of pain was seen in individuals with more chronic diseases, which in turn increased the likelihood of the individuals developing depression (OR = 2.777, P < 0.001, CI = 2.497-3.090) [18].

Likewise, between 2019 and 2020, Alhalal et al. carried out a cross-sectional study in Saudi Arabia on 233 chronic pain patients to examine the impact of chronic pain on depression [19]. They used the Chronic Pain Grade Scale to evaluate pain and the Center for Epidemiologic Studies-Depression scale to assess the patients' depressive symptoms. The result attained was that 36% of the patients examined had depression. Although it was determined that chronic pain intensity did not predict the presence of depression, pain disability showed a significant prediction of depression. For example, the worst depressive symptoms were seen in grade IV patients with a high amount of pain disability [19].

Studies have been conducted to test the relationship between chronic pain and depression, where models were created in rodents to observe the anxiodepressive-like outcomes of chronic pain [20]. The three chronic pain types that proved to be strongly associated with anxiodepressive-like conditions were: neuropathic pain, inflammatory pain, and fibromyalgia [20]. Arthritis is a condition that displays inflammatory pain; it was determined that 66% of individuals suffering from rheumatoid arthritis had been diagnosed with depression. Compared to other chronic pain conditions, anxiety and depression have been noted as the most commonly associated comorbidities of fibromyalgia [20]. A cross-sectional study using data from the Canadian Community Health Survey in 2000 and 2001 was conducted by Kassam and Patten [21]. The sample size was 115,160 participants aged 18 years or older. The outcome of the study showed that the prevalence of major depressive disorder (MDD) in patients with fibromyalgia (22.2%) was almost three times more than in subjects without the condition (7.2%) [21]. A population-based study with subjects from Denmark, the United States, and Spain showcased that patients with fibromyalgia had an elevated frequency of death due to suicide or suicidal attempts, and depression in these patients is strongly linked with suicidal thoughts and risks [22].

As age increases, the prevalence of chronic pain comorbidities is also expected to increase, which leads to more health concerns. A few studies have shown that 62.9-75% of chronic pain patients have at least one psychiatric disorder [23]. A Diagnostic and Statistical Manual of Mental Disorders (DSM)-IV (SCID)-based study, with a sample of 108 chronic pain patients and 54 control subjects, was conducted by Annagür et al. to identify Axis-I psychiatric disorders in patients with chronic pain [23]. The results showed that the prevalence of depression in chronic pain patients was 49.1%, which was also the psychiatric disorder most commonly associated with chronic pain [23]. Similarly, a cross-sectional study was carried out by Proctor et al. with 216 chronic pain patients from a neurodiagnostic clinic in the United States to estimate psychiatric disorder prevalence in chronic pain patients [24]. Symptoms were assessed through DSM-IV criteria as well. The study showed a prevalence of 44.4% for MDD [24]. As seen from the findings from the mentioned studies, the prevalence of depression in chronic pain patients is high. Hence, patients should be assessed for psychiatric disorders and treated accordingly.

Prevention of chronic pain

Chronic pain prevention is vital due to it being necessary to control the condition before the onset, which in turn can help prevent comorbidities that may occur along with chronic pain. There are various methods that can be used for chronic pain prevention [25]. Acute pain is the warning process that works to prevent tissue damage, but in some cases, pain can continue past the injury, becoming chronic pain. Primary prevention is one method used to prevent chronic pain; it involves the prevention of acute pain, in which one of the prevention processes is preemptive analgesia. In preemptive analgesia, peripheral or central sensitization is controlled, for example, through the sensitization of stimuli, which can be carried out by operative methods [26]. Another option would be through secondary prevention, where acute pain is identified early and treated aggressively, preventing chronic pain. Although peripheral sensitization has happened, the objective is to prevent central sensitization [26]. Multimodal analgesia is a method that is also used, where opioid and non-opioid analgesics are combined and administered to act on the pain pathway in different areas [26]. This process helps to improve pain control and has additive or synergistic effects. A narrative review article, which included 10 clinical studies, four surveys, and 10 reviews, aimed to showcase the extent to which physical activity could be preventative of chronic pain. It concluded that physical activity positively affects the prevention of chronic pain [27]. Although not all studies have shown only the positive effects of exercise on chronic pain, this inconsistency is varied chiefly based on the types of exercises used in each study. With exercise, levels of the adrenocortical hormone, cortisol, and catecholamine are higher, decreasing pain sensation [28]. If the prevention of chronic pain can be carried out effectively, comorbidities that occur along with it, such as depression, can also be prevented.

Management of chronic pain-induced depression

Drugs Used for Management

The most effective drugs for the treatment of chronic pain are opioids, which have been proven to be an effective mode of treatment for numerous chronic pain conditions such as cancer, nociceptive and neuropathic pain [11]. Pain is relieved through the combination of opioids with opioid receptors [11]. Although it has been shown that opioids are an effective form of treatment for chronic pain, their role in antidepressant therapy is still being studied [11]. Based on research, it has been proven that there are three types of classical receptors: μ, δ, and κ. These receptors are involved in mood regulation, and receptor κ has been seen to provide an antidepressant effect [11]. Studies have shown that buprenorphine can be used to treat refractory depression in middle-aged and elderly patients because it is an antagonist for the κ receptor and, in turn, has a good affinity for the δ opioid receptor [11]. Although studies have established that opioids can be used to treat chronic pain-induced depression, there has been a controversy that suggests that the use of opioids for long periods actually increases the risk of depression [11]. A cohort study conducted by Scherrer et al. used data from three American health systems (Veterans Health database, Baylor Scott and White Health, and the Henry Ford Health System) [29]. The results from the Veterans Health database showed an 18% increase in risk for depression in patients taking opioids for 31-90 days compared to patients taking opioids for 1-30 days [29]. Another group of analgesic drugs that have a therapeutic effect on chronic pain is benzodiazepines. They are involved in the antihyperalgesic effect of the GABAA receptor that is a target for benzodiazepines in the spinal cord, along with which the GABAA receptors have been seen to play a role in the regulation of mood and antidepressant therapy [11]. Along with treating chronic pain-induced depression, benzodiazepines are also used for treating anxiety and insomnia, both of which can be seen in chronic pain patients [30].

Due to increased prescription of the drugs, misuse has become more prevalent, especially in the recent coronavirus disease 2019 (COVID-19) pandemic, which led to increased social distancing, isolation, and loneliness, all of which contribute to depression, anxiety, or insomnia [30]. The motivations for misusers have been to help with sleep, experimentation, and to get "high" [30]. The methods for obtaining benzodiazepines have been primarily from family or friends compared to doctors [30]. Long-term cognitive disorders and addiction have been seen as the consequences of misuse [30]. Gabapentanoids have also been seen to be given in combination with opiates for treating chronic pain, as pregabalin is seen to promote the effects of opiates and decrease withdrawal symptoms [31]. On the contrary, the adverse effects of this combination have been respiratory and central depression [31]. As a result, managing chronic pain-induced depression should include physical exercise and psychotherapy, not be limited to pharmacotherapy, and patients being prescribed pregabalin should be closely monitored [31].

Antidepressant drugs have also been proven to be an effective mode of treatment for chronic pain-induced depression [11]. Monoamine oxidase (MAO) can be classified into type A (norepinephrine (NE), 5-hydroxytryptamine (5-HT)) and type B (phenylethylamine, benzydamine). MAO plays a role in the amine degradation pathway and is a crucial enzyme in the course. Type A MAO is seen to be more involved in developing mental disorders. A decrease in NE and 5-HT levels is suggestive of clinical depression at which monoamine oxidase inhibitor (MAOI) can be used as a treatment to increase levels at the appropriate sites. Moclobemide is an MAOI that has been used recently due to its effect on chronic pain and its ability to inhibit type A MAO providing an antidepressant effect [11]. A study conducted by Rowbotham et al. with 47 neuropathic pain patients in a clinical trial showcased the comparison of three antidepressants (amitriptyline, fluoxetine, and desipramine) for their effectiveness in chronic pain [32]. The results showed that desipramine and amitriptyline provided proper pain relief for 53-80% of the trial participants [32]. Hence, tricyclic antidepressants such as nortriptyline and desipramine have been used for the treatment of chronic pain more in recent times due to pain and depression having similarities in their neuroplastic changes [11]. They work to inhibit the reuptake of 5-HT and NE at the synapse junction and help inhibit endogenous pain in the central nervous system [11]. Although an ideal antidepressant hasn't been identified yet, serotonin reuptake inhibitors (SSRIs) are better tolerated by patients than other antidepressants [33]. An open-label study conducted by Shimodozono et al. involved using fluvoxamine in treating central poststroke pain, where the drug was given to 31 patients [33]. The results also showed significant improvements in the VAS and the Zung Self-rating Depression Scale [32]. Ferreira et al. reviewed 33 randomized controlled trials that were carried out to test the efficacy of antidepressants for back pain [34]. These trials involved 5318 participants, and serotonin-norepinephrine reuptake inhibitors (SNRIs) were administered. The results showed moderate evidence that SNRIs decreased back pain [34].

Psychotherapy

**Figure 1:**
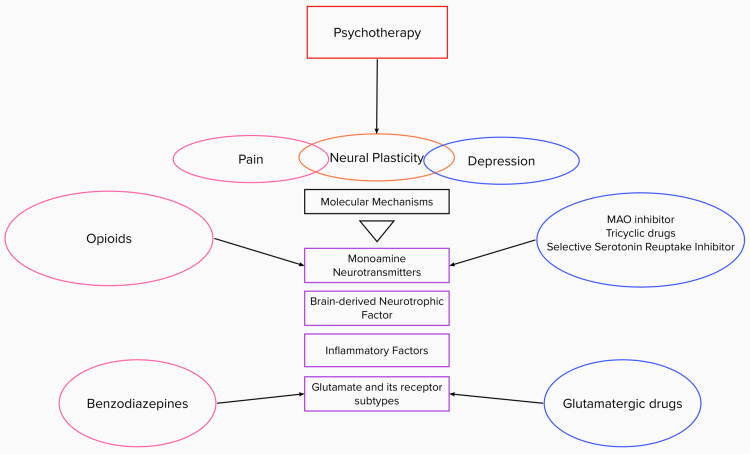
Treatment for chronic pain-induced depression MAO: monoamine oxidase Image credit: Roja Meda

The development of chronic pain-induced depression is also highly influenced by psychosocial factors. Eccleston et al. conducted a retrospective study involving 27 clinical trials, which proved that treatment with psychotherapy in adolescents and children with chronic headaches reduced pain [11]. The application of psychotherapy, such as cognitive-behavioral therapy (CBT), plays a crucial role in the treatment process [11]. It is a method used to help patients cope with emotional distress and limitations in function caused by painful conditions [35]. CBT has been proven to help conditions such as fibromyalgia, improve quality of life and functional status, as well as improve chronic pain levels [35]. In a randomized clinical trial conducted by Cherkin et al., 342 patients between the ages of 20 and 70 with chronic back pain were given CBT compared to the usual treatment that would have been given [36]. The results showed that the patients had a much more significant improvement in function [36]. This indicates that psychotherapy helps improve patients' prognosis and should be given as adjuvant therapy (Figure 1).

Cognitive functional therapy (CFT) is another method to help patients cope with chronic pain-induced depression [35]. CFT differs from CBT in that it showcases abnormal behaviors in a direct manner and challenges patients to alter them through their thought processes progressively and functionally. Four methods are used to modify the response of an individual to pain: making sense of pain, functional integration, exposure with control, and lifestyle change [35]. When these methods are implemented, a lifestyle change is seen in patients suffering from chronic pain. Some changes include regulating sleep cycles, avoiding sedentary actions, and exercising regularly [35]. Both CFT and CBT are patient-compliant due to the adaptable nature of the therapies based on the patients' needs [35].

Table 3 showcases details of studies involving the prevalence and treatment of chronic pain-induced depression.

**Table 3:** Summary of studies exploring the prevalence and treatment of chronic pain-induced depression SNRIs: serotonin-norepinephrine reuptake inhibitors; BrdU: bromodeoxyuridine; CBT: cognitive behavioral therapy; MDD: major depressive disorder; HDAC: histone deacetylase; VAS: visual analog scale

Reference	Study Type	Population	Conclusion
Alhalal et al. (2021) [19]	Cross-sectional study	233 chronic pain patients	Pain disability showed a significant prediction of depression, 36% of the patients had depression
Ma et al. (2021) [18]	Cross-sectional study	15,213 individuals from the 2015 China Health and Retirement Longitudinal Study	Increased severity of pain increased the likelihood of the individuals developing depression
Ferreira et al. (2020) [34]	Systematic review	5318 participants	Moderate evidence that SNRIs decreased back pain in patients
Schäfer et al. (2020) [27]	Narrative review		Physical activity has a positive effect on the prevention of chronic pain
Yalcin and Barrot (2019) [22]	Review	Population of Denmark, United States, and Spain	Elevated frequency of suicidal deaths in patients with fibromyalgia
Duric and McCarson (2019) [13]	Randomized control trial	Rodents	Reduction in BrdU positive cells in rodents after exposure to long periods of stress
Fisher et al. (2017) [37]	Review	27 clinical trials with children and adolescents	Psychotherapy reduced chronic headache in children and adolescents
Cherkin et al. (2017) [36]	Randomized clinical trial	342 patients between 20 and 70 years	Patients given CBT had greater improvement in function
Scherrer et al. (2016) [29]	Cohort study	Three American health systems’ data (Veterans Health database, Baylor Scott and White Health, and the Henry Ford Health System)	18% increase in risk for depression in patients taking opioids for 31-90 days than patients taking opioids for 1-30 days
Descalzi et al. (2015) [3]	Review	Mice	Removal of HDAC5 reduced depression-like behaviors in mice
Dureja et al. (2014) [16]	Epidemiological study	5004 respondents from India	Patients with chronic pain found it difficult to keep an independent lifestyle
Annagür et al. (2014) [23]	SCID-based prospective study	108 pain outpatient clinic patients at the Selcuk University aged 18-56 years	49.1% prevalence of depression in chronic pain patients
Proctor et al. (2013) [24]	Retrospective cross-sectional study	216 chronic pain patients in a neurodiagnostic clinic in the United States	44.4% prevalence of depression in chronic pain patients
Kassam and Patten (2006) [21]	Cross-sectional study	115.160 Canadian adults	Prevalence of MDD in patients with fibromyalgia was almost three times more than in subjects without it
Rowbotham et. Al (2005) [31]	Randomized clinical trial	47 neuropathic pain patients	Pain relief was seen in 53%-80% of the participants
Bair et al. (2003) [7]	Literature Review	Chronic pain patients from various clinics	Increase of lifetime prevalence of depression in chronic pain patients from 12% to 32% and from 32.4% to 56.8%
Shimodozono et al. (2002) [33]	Open label study	31 patients with central poststroke pain	Significant improvement in the VAS and Zung Self-Rating Depression Scale in the patients

Limitations

Some studies reviewed in the article are not from recent years; hence, interpretations of results and inferences may vary. Additionally, not all forms of treatment options and methods of prevention for chronic pain have been discussed due to the focus of this article being on chronic pain-induced depression.

## Conclusions

Chronic pain-induced depression is a prevalent condition that should have increased awareness. In summary, this review article aimed to provide an understanding of chronic pain and showcase the effect depression has on this condition. We have discussed the importance of the prevention of chronic pain where comorbidities that are seen along with it, such as depression, can also be controlled. Management options have been shown, suggesting the use of drugs, exercise, and psychotherapy, which are crucial methods for coping with this condition. This article provides the necessary information needed to understand the course of how chronic pain develops and, in turn, causes depression. Additionally, the molecular mechanisms through which both chronic pain and depression work have been shown, along with drugs that act through these mechanisms, such as analgesics. We believe that this article can serve as a guide for further understanding chronic pain-induced depression and help with its prevention to decrease its prevalence. As discussed, the prevention of chronic pain can be carried out through the methods of primary and secondary prevention, depending upon the severity. As the world is becoming more open-minded about the importance of mental health, this article aims to further aid the process through this discussion on chronic pain-induced depression and help bring about a better understanding of those suffering from the condition. As this is a topic that is becoming more prevalent, we recommend future studies to keep up with forthcoming advances and changes.
